# Advances in Microbiome-Derived Solutions and Methodologies Are Founding a New Era in Skin Health and Care

**DOI:** 10.3390/pathogens11020121

**Published:** 2022-01-20

**Authors:** Audrey Gueniche, Olivier Perin, Amina Bouslimani, Leslie Landemaine, Namita Misra, Sylvie Cupferman, Luc Aguilar, Cécile Clavaud, Tarun Chopra, Ahmad Khodr

**Affiliations:** 1 L’Oréal Research and Innovation, 93600 Paris, France; audrey.gueniche@rd.loreal.com (A.G.); olivier.perin@rd.loreal.com (O.P.); leslie.landemaine@rd.loreal.com (L.L.); namita.misra@rd.loreal.com (N.M.); sylvie.cupferman@rd.loreal.com (S.C.); luc.aguilar@rd.loreal.com (L.A.); cecile.clavaud@rd.loreal.com (C.C.); 2 L’Oréal Research and Innovation, Clark, NJ 07066, USA; amina.bouslimani@rd.loreal.com; 3 L’Oréal Research and Innovation, Singapore 048583, Singapore; tarun.chopra@rd.loreal.com

**Keywords:** skin health, microbiome, postbiotics, microbiome metabolites, cosmetic, microbiome data, methodology harmonization

## Abstract

The microbiome, as a community of microorganisms and their structural elements, genomes, metabolites/signal molecules, has been shown to play an important role in human health, with significant beneficial applications for gut health. Skin microbiome has emerged as a new field with high potential to develop disruptive solutions to manage skin health and disease. Despite an incomplete toolbox for skin microbiome analyses, much progress has been made towards functional dissection of microbiomes and host-microbiome interactions. A standardized and robust investigation of the skin microbiome is necessary to provide accurate microbial information and set the base for a successful translation of innovations in the dermo-cosmetic field. This review provides an overview of how the landscape of skin microbiome research has evolved from method development (multi-omics/data-based analytical approaches) to the discovery and development of novel microbiome-derived ingredients. Moreover, it provides a summary of the latest findings on interactions between the microbiomes (gut and skin) and skin health/disease. Solutions derived from these two paths are used to develop novel microbiome-based ingredients or solutions acting on skin homeostasis are proposed. The most promising skin and gut-derived microbiome interventional strategies are presented, along with regulatory, safety, industrial, and technical challenges related to a successful translation of these microbiome-based concepts/technologies in the dermo-cosmetic industry.

## 1. Introduction

Applications of Microbiome sciences are very large and have been proposed as a potential target solution for the 21st century socio-economic and environmental challenges [1]. For several decades, scientists have been interested in the microbiome and its impact on human health. A major focus was put on the gut microbiome, and its role in human health has been well established [2]. New knowledge on lung, oral cavity, and skin microbiome is beginning to emerge [3]. A deeper knowledge of the microbiome, specifically that of the skin, opens perspectives for a revolution in dermo-cosmetic development. These recent discoveries have changed our perception of the role of bacteria in skin health. For example, microbiome-derived and personalized dermo-cosmetic development would be possible, due to the advancement in skin microbiome analysis and diagnosis [4]. New products that respect, protect or rebalance the skin microbiome are a new trend in the dermo-cosmetic industry.

The main aim of this review is to share a global view covering mechanistic knowledge regarding the interaction between the microbiome (gut and skin) and the skin. It also focuses on how this knowledge is translated into products/concepts in the dermo-cosmetic field. The latest methodological developments for skin microbiome analysis and the specific regulatory/safety environment of microbiome-derived solutions used in the dermo-cosmetic industry are summarized. The translational challenges of the presented microbiome-based concepts are also discussed.

## 2. The Skin and Its Microbiome

In adults, considering the appendages, the skin forms a large surface of 30 m^2^ [5]. This extensive surface constitutes an important protective barrier that is physically ensured by epithelial cells and is also ensured by the presence of a beneficial microbiome that interferes with the three previously stated barrier properties.

The microbiome is an essential partner to our skin. It is a beneficial and invisible ecosystem of living microorganisms that is an integral part of our skin’s surface. It is a natural ecosystem of microbes that protects our skin from external damage and acts as a second genome, interacting with our bodies to ensure healthy functioning. It plays a major role in our skin defense and regulates the exchanges between the body and the environment. Its balance is essential to our skin health and beauty.

The composition of the skin microbiome varies according to body sites that constitute diverse ecological/physicochemical niches. Briefly, the skin can be moist, dry, or sebaceous, and each of these classes has a distinct subset of microbial taxa that are particularly well suited to these conditions. Lipid content is a key factor driving the microbiome composition that drastically changes between dry and sebaceous sites [6,7]. On sebaceous sites lipophilic *Cutibacterium* species (spp.) are the most abundant, whereas bacteria such as *Staphylococcus* and *Corynebacterium* spp. are preferentially abundant in moist areas, *Cutibacterium* spp., *Staphylococcus* spp., and *Streptococcus* spp. are the most abundant bacteria on dry sites, [8,9]. *Malassezia* spp. is the most abundant fungi across the body, except for the sites on the foot which present greater diversity. *Cutibacterium acnes* (*C. acnes)* phage is the most represented virus in different skin sites and eukaryotic viruses are more transient [9,10]. *Cutibacterium* spp. and *Staphylococcus* spp. play important and multiple roles on the skin.

*S. epidermidis* is traditionally considered to be one of the major representative bacteria of healthy skin Microbiome. It is among the most abundant species of the cultivable microbiome: up to 90% of the cultivable aerobic flora [11]. It is ubiquitous: present on the whole body in dry, wet, and sebaceous areas [12]. Recent studies have shown its role in maintaining an effective skin barrier in vitro [13,14], in wound healing [15]; in the fight against pathogens [16,17]; in modulating the immune system [15,18,19,20,21], and in preventing melanoma [22].

*C. acnes* is also a highly represented bacterium in the human skin microbiome (>50% of bacterial species according to sequencing data), about 10^5^ bacteria/cm^2^ on sebaceous areas (face, scalp, back; rich in sebum). *C. acnes* degrades long-chain fatty acids in sebum to short-chain fatty acids (SCFA), including propionic acid (or propanoic acid) via its lipases activity [11]. Propionic acid is involved in skin odor, but more importantly, it maintains the pH of the skin and has antimicrobial properties [11]. *C. acnes* also participates in antimicrobial defenses through the secretion of bacteriocins or antimicrobial peptides like cutimycin [23,24]. It has been associated with and is believed to play a major role in skin health. It synthesizes free fatty acids such as vitamin B12 which deficiency can be associated with skin hyperpigmentation, vitiligo, peeling [25]. Vitamin B12 biosynthesis is decreased in acne patients compared to healthy individuals [26]. Conjugated linoleic acid (anti-proliferative), riboflavin (vitamin B2, antioxidant [27] and folate (vitamin B9, play a role in cell division and may protect the skin barrier especially following sun exposure [28,29,30] are other examples of the implication of *C. acnes* in skin health.

The interaction between *S. epidermidis* and *C. acnes* postbiotics/metabolites and skin health is detailed in the upcoming “effector molecules/metabolites” section.

Among the other bacteria, some have been described for their beneficial effects on the skin, for example, *Corynebacterium* spp. which allows the regulation of the immune system [31], *Micrococcus luteus* degrade pollutants and isomerizes urocanic acid, which may play a role in UV protection [32,33].

Compared to other tissues, the skin microbiome shows the highest individual diversity and is less stable [34]. This is not surprising because of the exposure of the skin microbiome to external factors (i.e., UV, humidity, pollution, environmental bacteria, cosmetics). The impact of external factors (exposome) on the microbiome has been comprehensively reviewed by Skowron et al. and is addressed hereafter [35].

Despite these extrinsic factors, samples and data generated from longitudinal studies show that the skin microbiome is, to some extent, stable at a strain level. Nevertheless, stable specific individual signatures at a strain or SNV level exist. This is the case for *S. epidermidis* on some specific sites [7,36]. This stability is not contradictory with the assumption that transient microbiome can enter the community from the environment (home, pets, other individuals).

Overall, the skin harbors a stable and diverse community of microorganisms that interact with the external environment, skin cells, and other microbial cells to maintain its homeostasis. Any dysbiosis driven by the overabundance of one of the commensal opportunistic microorganisms can be associated with skin diseases/conditions.

## 3. Skin/Scalp Conditions and Microbiome

The skin microbiome plays an important role in maintaining cutaneous health with the skin microflora constantly adapting in response to intrinsic and extrinsic factors. The environment, and therefore pollution exposure, has the potential to influence the skin microflora and bacteria isolated from the human skin have been shown to degrade Polycyclic Aromatic Hydrocarbons (PAHs) and related xenobiotic compounds [37].

### 3.1. Variability across Age

Today, a widely accepted assumption is that the environment of the fetus is sterile, and that colonization of the skin occurs at birth. This first microbiota varies, depending on the mode of delivery: a vaginal birth allows colonization of the baby’s skin by the mother’s vaginal microbiota (mainly *Lactobacilli*), while a C-section is associated with the colonization by bacteria from the operating room environment and the mother’s skin (*Staphylococcus* spp., *Corynebacterium* spp., *Cutibacterium* spp.) [38]. The difference in microbiota acquired at birth may have a longer-term impact on the microbiota that will develop in childhood [39]. The low microbial diversity and the non-exposure to vaginal bacteria in children born by cesarean section could cause a delay in the development of their immunological functions which could partly explain their greater sensitivity to certain pathogens and allergies, and an increased risk of developing atopic dermatitis in childhood [40,41,42,43].

The microbiota changes gradually during the child’s first year. Unlike the adult individual, the skin microbiota of newborns is homogeneous throughout the body, regardless of the type of delivery [44]. Primary colonization is characterized by a high proportion of *Staphylococci* which will gradually decrease as the microbiota is enriched with new bacterial populations. The development of this diverse microbiota, from the first months of life onwards, with regards to the maturation of the skin, in particular its immune system, contributes to the establishment of skin homeostasis [45]. The diversity and composition of the microbiota remain stable during childhood.

At puberty, sebum secretion increases and promotes colonization by lipophilic bacteria and in particular *C. acnes*, involved in acne [46]. Their population begins to increase at the age of 10 years, but it is especially between 15 and 25 years that studies have measured the strongest expansion of these bacteria [47,48]. The increased secretion of sebum also affects populations of fungi. The fungal diversity observed in children decreases with age and lipophilic fungi are favored: the proportion of *Malassezia* increases and becomes dominant, in particular, *M. restricta* in sebaceous areas [49].

**Figure 1:**
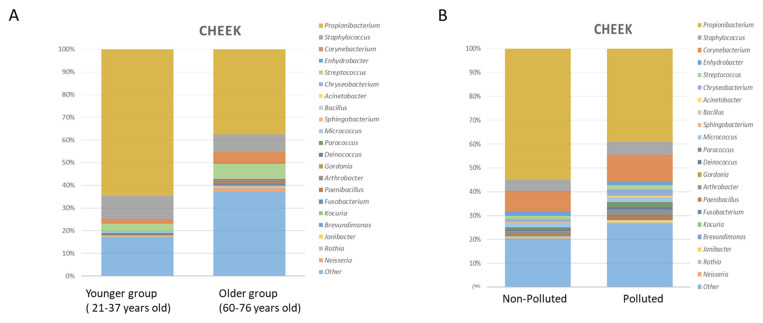
(**A**) Taxonomic analysis of cheek microbiome in younger and older subject’s group. Stacked bar charts showing the relative abundance of the 20 most prevalent bacterial genera. Adapted from [56] (**B**) Taxonomic analysis of cheek microbiome (younger group) in polluted and non-polluted environment. Stacked bar charts showing the relative abundance of the 20 most prevalent bacterial genera. Adapted from [57].

Between around 25 and 60 years of age, the microbiota is stable with equivalent environmental factors [7]. With age, from 55–70 years, depending on the population, the microbial diversity increases [50,51,52,53,54]. In addition, a significant decrease in *C. acnes* is observed, associated with a decrease in sebum secretion and an increase in skin dryness [55,56]. Variation of the Microbiome according to age is resumed in Figure 1A. This difference is accentuated among centenarians [53].

### 3.2. Variability Due to Extrinsic Factors

Skin microbiota modifications are also associated with extrinsic factors, such as urbanization, exposure to antibiotics, air pollution, UV exposure, smoking, hygiene products [57,58,59,60,61,62,63,64]. The set of factors to which an individual and the skin microbiota are exposed is called the exposome [65].

The modulation of cosmetic formulas on the microbiome is still under investigation and might be formula and skin site-specific. On one hand, some major components of the facial microbiome decrease after cosmetic use [66]. Furthermore, multiple studies suggest a strong effect of antiperspirant and deodorant use on the bacterial composition of armpits [67]. On the other hand, formulas used in the cleansing axis had a strong impact on the microbiome, even though some abundant taxa have been shown to be resilient [68]. Conversely, arm and face lotions had little effect on bacterial and archaeal diversity [69]. Finely chosen preservative systems for cosmetic formulas have been shown to preserve the homeostasis of the microbiome and even help to restore its equilibrium after dysbiosis [70]. In another study, the authors showed that although tested preservatives had an efficient antibacterial activity in vitro, the skin microbiome was not impacted in vivo [71]. Cosmetic serum-containing galactooligosaccharides (GOS) prebiotics increased the Shannon index diversity post-treatment in the experimental group vs. control. *S. aureus* relative abundance decreased. Changes in the Microbiome composition were associated with improvement in various clinical parameters (e.g., water loss, wrinkles depth) [72].

The impact of microbiome modifications on skin health in such conditions is still unclear, however, some recent evidence has been reported in the case of pollution using microbiome and metabolomics data of a Chinese cohort [57]. The impact of pollution on the Microbiome composition is resumed in Figure 1B. Comparing skin microbiota and skin metabolome allowed the authors to highlight a strong link between sebum degradation and bacterial taxa (*Cutibacterium* spp. and *Staphylococcus* spp.) and revealed a potential link between these taxa and the modulation of metabolites such as carnitine, histamine, and phenyl-lactic acid (PLA) on the skin, which could represent new factors involved in the commensal-host homeostasis [73].

### 3.3. Variability Associated with Skin Health and Disorders

The skin microbiome associated with skin conditions has been observed in many skin disorders such as atopic dermatitis (increase in the proportion of *S. epidermidis* and *S. aureus*), psoriasis (increase in the proportion of *S. aureus*), acne (increase in the proportion of particular phylotypes of *C. acnes*), seborrheic dermatitis and dandruff (increase in the amount of *Malassezia*), vitiligo (decrease in microbial diversity and increase in the proportion of Firmicutes at the lesion level [74]. The association between the skin microbiome composition and skin health/conditions is resumed in Table 1.

The features of the skin microbiome in common inflammatory skin diseases have been reviewed earlier [95]. Dysbiosis of the Microbiome is observed in many skin conditions such as atopic dermatitis, acne, seborrheic dermatitis and dandruff (increased amount of *Malassezia*), vitiligo (decrease in microbial diversity and increase in the proportion of Firmicutes in the lesions) [74]. Minor species represent 5 to 20% of bacteria and fungi and up to 1000 different species have been identified. Their proportion and diversity vary according to the anatomical zones, the type of skin, and various factors (i.e., environmental, age, lifestyle hygiene). Their precise role remains to be elucidated [96]

#### 3.3.1. Role of *S. epidermidis*

In Atopic dermatitis, a dysbiosis of the skin microbiome is observed with an increase in the proportion of *S. aureus*, and *S. epidermidis* during the atopic crisis [75,97,98]. *S. aureus* is considered as an opportunistic pathogen, as various virulence factors have been described (formation of biofilms, toxins, Phenol-soluble modulins (PSMs), proteases) in association with skin disorders [99,100,101]. It is a coagulase-positive bacteria, unlike the majority of *Staphylococci* found on the skin, which are coagulase-negative *Staphylococcus* (CoNS: Coagulase-negative *Staphylococci*) [84].

Initially, the increase in *S. epidermidis* was described as a defense against *S. aureus* proliferation (via Esp protease which inhibits biofilm formation by *S. aureus* [102] and via secretion of lantibiotics [103]), but new data on *S. epidermidis*/skin interaction in a mouse model suggests that *S. epidermidis* may have a pro-inflammatory role and alter the skin barrier in the context of atopic dermatitis and Netherton syndrome [104,105]. It has also been shown that excessive colonization of the skin by *S. epidermidis* could lead to an alteration of the cohesion of the epidermis following the lysis of desmosomes by the bacterial proteases EcpA (Extracellular cysteine protease A) [104]. Further studies are required to unambiguously demonstrate the role of *S. epidermidis* in atopic dermatitis.

#### 3.3.2. Role of *C. acnes*

The major microorganism of the pilosebaceous unit is *C. acnes*, representing up to 90% of the microbiome in sebum-rich sites such as the scalp, face, chest, and back. Numerous paths have been proposed by which *C. acnes* exacerbates acne, including augmentation of lipogenesis, comedone formation, and host inflammation [106]. *C. acnes* promotes comedogenesis by generating oxidized squalene and free fatty acids, leading to a qualitative change in sebum [107,108]. Moreover, *C. acnes* activates the IGF-1/IGF-1 receptor signaling pathway to upregulate filaggrin expression, which in turn increases integrin-α3, -α6, and vβ6 levels, thereby affecting keratinocyte proliferation and differentiation and resulting in comedone formation [109,110]. It has been shown to trigger sebum accumulation when applied to hamster auricles [111]. In addition, *C. acnes* induces and aggravates inflammation, by activating Toll-like receptors (TLR-2 and TLR-4) on keratinocytes, which leads to the activation of the MAPK and NF-kB pathways [112,113]. Additionally, metabolites such as porphyrins produced by *C. acnes*, are pro-inflammatory and are associated with acne disorders [114]. Strain-level differences in porphyrin production and regulation in *C. acnes* elucidate disorder associations [115]. It is suggested that indole compounds produced by *Malassezia* spp. downregulate the inflammatory response, thereby helping to establish the associated pathology *Pityriasis versicolor* [116].

#### 3.3.3. Role of Other Microorganisms

*C. acnes* is not the only microbial player in acne. Intriguingly, according to data from Barnard et al. and Park et al., the abundance of the genus *Cutibacterium* is slightly higher on healthy skin compared to acne-affected skin [117,118]. Compared to healthy controls, an increase of *Cutibacterium granulosum*, *S. epidermidis*, *Proteobacteria*, *Firmicutes*, *Streptococcus* (pre-adolescent) and *Malassezia* species associated with a decrease in *Actinobacteria* has been also described. An increase in Bacteroides in the gut Microbiome has been also observed [106]. In comedones and pustules, *C. granulosum* known for its higher lipase activity is highly abundant [109]. This high lipase activity (100 times higher than *C. acnes*) is also the major driver for the implication of *M. restricta* in acne pathology in young adults with refractory acne [106]. In summary, the direct involvement of the microorganisms or their interaction with *C. acnes* still needs to be elucidated. Dissection of the role of *C. acnes* in acne is further complicated by recent findings that suggest that specific *C. acnes* phylotypes could play a major role in acne aggravation, while others could be beneficial for the skin.

In Psoriasis, Microbiome is affected. However, no clear conclusion is established that connects the Microbiome diversity to the disease status. Different studies show that there is either an increase or a decrease or no change in the microbiome diversity [95]. Nevertheless, there is evidence that shows a higher *S. aureus* and a lower *S. epidermis* abundance in psoriatic lesions [119,120].

More importantly than the connection between microbiome composition and psoriasis or any other skin condition, it would be of interest to gain a better understanding of the functional relation between the Microbiome and these different skin conditions. Nonetheless, Microbiome modulation could be an attractive therapeutic approach. Thus, ingredients/products that modulate beneficially the Microbiome could help to improve the clinical signs associated with each skin condition and is well covered in the review by Polak et al. [121]. Many topical or oral-formulas show improvement of atopic and acneic skin conditions after their application/ingestion. Topical formulas modulate the skin Microbiome mainly via their antibacterial activity against *C. acnes* and/or *S. aureus* the two major implicated microorganisms [121,122,123].

Different confounding factors could likely explain these controversial conclusions about Microbiome diversity among the different studies trying to establish a Microbiome signature in each skin condition (cosmetic routines, exposome, skin site…) but one of the most important factors explaining these disputed results could be the diverse approaches for skin Microbiome analysis from wet lab to bioinformatics.

The study design for skin microbiome research is multifaceted and integral to all downstream steps. Many published studies examined the biases introduced by the skin sampling methods and sample storage, controls and contamination sources, sequencing biases, and possible quantitation [124,125,126,127,128,129]. The complexity of skin microbiome studies is summarized by Kong et al. [130].

Adapted methodology strategy dedicated to the skin microbiome and its metabolite analysis is key for a successful translation into microbiome-based concepts. Standardization and robustness of the tools are of great importance, in addition to the use of a collection of cutting-edge technologies which aim to reduce the bias (mock communities, primers choice adapted for skin microbiome analysis, distinguishing between dead/alive bacteria for accurate estimation of diversity, platform methods and metadata validation/standardization…).

## 4. Methods in Skin Microbiome Exploration

Over the last two decades, the development of the so-called Next Generation Sequencing (NGS) technologies and their continuously decreasing costs have allowed the generation of a huge amount of metagenomic data. Today, these data represent a strong lever for microbiota characterization, whether in healthy conditions or disorders, including infectious diseases, cancers, and other different clinical applications [131,132,133,134,135,136]. In dermatology, for example, multi-omics have increased the knowledge of many skin disorders, such as psoriasis, atopic dermatitis, or acne [76].

### 4.1. Perspectives for Skin Microbiome Metabolomics Analysis

An emerging “omics” tool to understand the function of the human microbiome is metabolomics (i.e., the analysis of small molecules including sugars, amino acids, lipids, and nucleotides, in a system) [137]. In gut microbiome studies, metabolomic tools have been extensively applied to characterize microbial metabolites and to determine their role in mediating host-microbe crosstalk [138,139]. Particularly, untargeted metabolomics (i.e., metabolomic profiling to collect a chemical inventory from a sample) has led to the discovery of many microbiome-derived biomarkers of host diseases, such as inflammatory bowel syndrome, Crohn’s disease, diabetes, and non-alcoholic fatty liver disease [140,141,142,143]. These metabolomic applications have led to the identification of important bacterial-based targets for disease diagnosis and treatment, inspiring the use of metabolomic approaches to characterize the molecules associated with the skin microbiome and to explore their role in influencing skin physiology [144].

In dermo-cosmetics, understanding the relationships between the chemistries involved in host-microbiota-environment crosstalk is crucial for the development of innovative microbiome-based solutions to treat skin conditions. The development of new sampling methods for the collection of skin samples compatible with untargeted metabolomic workflows has greatly improved the characterization of the chemical composition of the human skin [144,145,146,147]. Although the identification of microbial metabolites from skin samples remains challenging, mainly due to the underrepresentation of reference spectra collected from microorganisms in public libraries, integrating metabolomics and microbiome data has improved our ability to identify microbe-metabolite associations. Since its development in 2015, the 3D cartography approach has become a powerful tool to visualize and integrate large-scale microbiome and metabolomics data from the skin surface and to rapidly screen for omic spatial distributions associated with different phenotypes, through spatial correlations between molecular and bacterial distributions [147]. This tool has also enabled the characterization of antimicrobial peptides (Human neutrophil Peptides—HNP-1 and 2) that co-localize with bacteria including *Provotella* and *Clostridium* in the groin area, as well as products of bacterial processing on the skin, such as free fatty acids resulting from hydrolysis of triacylglycerides mediated by *Cutibacterium*. As the tool continues to evolve, future applications to skin conditions, such as atopic dermatitis and psoriasis, may reveal biogeographical microbe-metabolite correlations differentiating healthy skin from diseased skin or lesional vs. non-lesional sites.

Recent advances in bioinformatics and computational pipelines have greatly facilitated the visualization and annotation of large-scale metabolomic data. Molecular networking, initially introduced in 2012, has become a powerful bioinformatics tool to organize, mine, and compare spectral data, as well as to connect related molecules by their spectral similarities [148]. This tool is part of an online infrastructure—GNPS (Global Natural Products Social Molecular Networking http://gnps.ucsd.edu, accessed on 25 October 2021) and enables the processing of large-scale metabolomics data and molecular annotation through the interrogation of reference spectral libraries [149]. Furthermore, multivariate analysis and machine learning tools have recently been implemented in GNPS to compare molecular profiles collected from different groups/types of samples [150]. Beyond its extensive application in natural product discovery, clinical and environmental studies, applications in skin studies included the identification of molecular signatures associated with individual lifestyles and further linked them to the built environment, investigating the impact of personal care products on the skin metabolomics and microbiome dynamics or even monitoring drugs and their metabolites directly on human skin [69,151,152,153,154,155,156,157,158]. Molecular networking has also proven to efficiently assign the origin of molecules detected on human skin (skin cells, microbes, environment, and lifestyles) [147,156,159]. Since the development of molecular networking, several complementary bioinformatic tools have been developed to enhance quantitative metabolomics analysis, expand molecular annotations through a chemical tree-based approach and in silico annotation tools, as well as to re-analyze public metabolomics data using formatted metadata to compare metabolites between groups of samples from different datasets [160,161,162,163].

Additional bioinformatic advances have improved functional interrogations of the human microbiome including genome mining tools, such as antiSMASH, SMURF, and PRISM that has greatly facilitated the prediction of biosynthetic gene clusters (BGCs). Further development of computational algorithms applied to metagenomic sequencing data of the human microbiome has led to the discovery of small molecule-producing BGCs, such as Type II Polyketides that appear to be widely encoded in the oral, gut, and skin microbiome [164,165,166,167]. Advances in algorithms that pair metagenomic and metabolomic datasets, such as NRPquest, NRPminer, and MetaMiner, have allowed more accurate identification of bioactive metabolites produced by BGCs from different environments [168,169,170]. Examples include the discovery of known and unknown post-translationally modified peptides (RiPPs) from lichen and human microbiomes, and the identification of Non-Ribosomal Peptides (NRPs), such as lugdunin from *Staphylococcus* skin isolates and surugamides from soil datasets [168,169].

### 4.2. Expectations from Metagenomic and Data Science

Metagenomics is of particular interest for the dermo-cosmetic industry, with the microbiome representing a real natural reservoir for the discovery of new molecules of interest [171,172,173].

Recently, Liu et al. described a screening approach to analyze over 3000 human skin isolates to evaluate bacterial competition within the human skin microbiota [174]. The authors demonstrated that bacteriocin micrococcin P1 (MP1) from the *Staphylococcus hominis* strain led to reduced *Staphylococcus aureus* infection and accelerated closure of *S. aureus*-infected wounds, meaning that MP1 can be proposed as a candidate to develop a new approach against *S. aureus* infections. More interestingly, their results show that, beyond the generation of metagenomic experiments to characterize microbial communities at the taxa level, it is also important to model the whole ecosystem of the microbiota better, including microbes-microbes but also host microbes’ interactions. The latter presents system biology approaches, largely described as being the future of metagenomics and microbiome modeling for biomarker discovery and new pharmacology applications [12,174,175].

### 4.3. Meta-Omics and System Biology Approach

In this field, challenges in bioinformatics and data science remain to develop new omics data integration and analytical strategies for microbiome analysis [176]. This task is not easy, as an important amount of omics data can now be simply generated for both humans and microbes on different omics layers (e.g., genomics, transcriptomics, proteomics, and metabolomics). Researchers deal more and more with meta-omics approaches to assess the potential functions encoded by microbial communities and quantify the metabolic activities occurring within a complex microbiome.

In 2019, Zhang et al. discussed that functional meta-omics approaches could be one of the most promising strategies to facilitate the identification of microbes’ metabolic pathways specific to clinical phenotypes [177]. In a second step, the authors suggested that these metabolic pathways could be modulated through either supplementation of beneficial species, engineered probiotics/commensals, prebiotics, bacteriophages, or highly selective drugs to shift microbiome metabolic profiles closer to healthy conditions.

### 4.4. Network-Based Models’ Approach

Compared to the meta-omics approaches described above, which can be considered as data-driven approaches, network-based models represent another category of system biology approach for microbiome analysis. Network-based models are mainly represented by genome-scale metabolic models (GEMs), which have been the subject of many technical publications in recent years [178,179,180,181,182,183]. GEMs aim to reconstruct biological networks by assembling several biological pathways created using experimental data [184,185]. A reconstructed network describes gene-protein-reaction associations for all metabolic genes in an organism. Even if the reconstructed network approach was firstly described on the level of a bacterium, the same approach has been applied to humans and *Recon* is a well-known published human metabolic map [184,186,187,188].

When combined, the human metabolic network and microbe networks can provide an interactomic map between microbe metabolites and the human proteome. As an example, Magnúsdóttir et al. reconstructed networks for 773 members of the gut microbiota [189]. This resource, called AGORA, is compatible with the human metabolic network *Recon*, thus representing a powerful strategy to facilitate the study of host-microbiome interactions. Although these network-based models were mainly published to study gut microbiota, a very recent review described how the same approach can be transposed to skin microbiome functional analysis [180,189,190]. In addition, this metabolic-network approach can provide major advantages for precision medicine and personalized cosmetic purposes. Indeed, in 2017, Jens Nielsen explained that multi-omics data coming from clinical trials can be translated to GEMs to identify “reporter” metabolites or “sub-networks” that could be specific to a cluster of patients [179].

Multi-omics data analysis using a reconstructed network thus appears as a strong lever to understand microbiome functions more thoroughly, including microbes-microbes and host microbes interactions as well as the mechanisms of bacterial colonization that can lead to skin-specific diseases or disorders.

Improvements in these approaches are still widely expected by the scientific community as they are promising tools for the discovery of new biomarkers, as well as for the development of future active ingredients for both dermatology and cosmetics.

However, the amount of work to establish a network on the microorganism scale properly remains laborious [178,191]. To avoid such a challenge, another method has been developed to predict the metabolome using metagenomic sequencing data only [144,192,193,194,195]. More recently, Yin et al. published a comparative analysis of their tool with two other methods published in this domain [196]. To evaluate the performance of the three different algorithms, 900 microbiome-metabolome pairs of samples from six different studies of human diseases have been used for occurrence prediction (presence vs. absence) and metabolites considered as differentially expressed. As these prediction tools only use metagenomic data as input, they may be considered as a good compromise and a serious alternative solution to the generation and analysis of meta-omics and network reconstructions that are known to be more expensive and time-consuming strategies.

In conclusion, associating multi-omics technologies, statistical and computational analyses, and more advanced 3D skin models, will offer a promising opportunity to establish a microbiome-related skin condition causality and subsequently orient toward the cosmetic solution/ingredient.

Moreover, the microbiome is nowadays a significant target of personalized technologies by presenting interesting solutions for different skin conditions, such as dryness, aging signs, reactive or irritated skin [197,198]. Applied microbiome research offers a functional alternative path towards new solutions that might possess other levels of performance.

## 5. Microbiome-Based Cosmetic Solutions and Technologies

Many of the skin conditions described above are multifactorial, however, the microbiome is a key factor in skin disorders. The interplay between the microbiome and the skin is key for its homeostasis health. Intervening and finely modulating the microbiome to correct skin conditions described above is a rising field of research. These interventions are mainly realized by prebiotics, postbiotics, and probiotics, as well as microbiota transplant. The latter is still in its infancy phase for the skin. In cosmetic/dermatology applications, a focus concentrates on the first three paths.

The microbiome has been extensively studied and reported in the field of nutrition [199]. Although some definitions exist on the World Health Organization level, there are currently no available international guidelines regarding the definitions or terminologies applicable for cosmetic ingredients that work with the skin’s microbiome. Current definitions consider probiotics to be living microorganisms that must be ingested in a sufficient amount to have a positive effect on health that is not limited to the nutritional effects alone [200,201,202].

Prebiotics are a food ingredient that results in specific changes in the composition and/or activity of the gastrointestinal microbiota, thus conferring benefit(s) upon the host’s health [199].

Very recently, the International Scientific Association of Probiotics and Prebiotics (ISAPP) defined the scope of postbiotics as a “preparation of inanimate microorganisms and/or their components that confers a health benefit on the host”.

Postbiotics could be intentionally inactivated microbial cells with or without metabolites, or cell components that contribute to establishing host health benefits.

The gut is not the only site of action of postbiotics. They could also be administered on a host surface, such as in the oral cavity or on the skin [203].

The topic of the cosmetic microbiome was taken up in 2018 by the International Cooperation on Cosmetics Regulation (ICCR), a voluntary international group of cosmetic regulatory authorities and cosmetic industry trade associations from Brazil, Canada, Chinese Taipei, the European Union, Japan, the Republic of Korea, and the United States. They considered that new technologies exploring the relationship between the human microbiome and healthy skin were an area of increasing interest and the safety, quality, regulation, and potential development of international guidelines for products arising from these technologies would be a worthwhile topic for the ICCR.

In 2020 they developed a set of categories and descriptors that could be used to group and categorize microbiome-related products, their ingredients, and other relevant approaches, in a cosmetic/skin-relevant context [204].

These ingredients were divided into two main categories based on viability: viable (live or dormant)—encompassing only probiotics (based on biological origin), and non-viable ingredients. The non-viable ingredients were further divided into two sub-categories; prebiotics (by their intended action on the skin microbiota) and postbiotics (based on their biological origin) (Table 2).

Postbiotic products/ingredients belong to the non-viable category. Based on their biological origin, postbiotic ingredients (ferments, extracts, lysates, filtrates) share a common description: “*Non-viable ingredients comprised of inactivated microorganisms and/or soluble factors (products or metabolic by-products) released by live or inactivated microorganisms, added to a cosmetic product to achieve a cosmetic benefit at the application site, either directly or* via *an effect on the existing microbiota*” [204].

In cosmetics, postbiotics may be an alternative to the use of live whole microorganisms in probiotic form. To summarize the product entries, postbiotic ingredients were divided into three types:

“*Ferments, lysates, extracts, filtrates or any combination of these ingredients that are not living but which have been obtained by means of probiotic bacteria (Bacillus, Bifidobacterium, Lactobacillus, Lactococcus, Vitreoscilla, Streptococcus thermophilus, Leuconostoc) or fungi used primarily as fermentation facilitators (Saccharomyces, Candida bombicola, Kloeckera, Hansenula-Pichia, Aspergillus)*”:

*“Non-viable microorganisms (inactivated/heat-killed), mostly lactic-acid forming bacteria: Enterococcus faecalis, Lactobacillus (paracasei, casei, acidophilus), Lactococcus, or Vitreoscilla filiform”.*

*“Metabolic products/by-products (isolated) including bacteriocin extract, ectoin, succinic acid, lactic acid, hydrolyzed yogurt protein, sodium hyaluronate, and milk proteins”* [204].

### 5.1. Postbiotics

While both prebiotics and probiotics are either used alone or in combination as symbiotics focus on beneficial bacteria directly, postbiotics focus on downstream benefits to the host and can be categorized into 3 major categories: 1. Inactivated microbial cells as lysates, including cell wall components and the cytosol, 2. Exopolysaccharides, 3. Secreted molecules, including peptides, proteins (enzymes), and small metabolites.

Postbiotics, also referred to as functional ingredients, impart their benefits through a myriad of bioactivities that can include anti-inflammatory, immunomodulatory, antioxidant, anti-microbial, pro-differentiation, etc. It is important to note that these activities are effectuated through important host-microbiome interactions.

Classically, postbiotics have mainly been studied in the context of the gut microbiome, especially for their applications in early life nutrition, wherein an important role for short chain fatty acids (SCFAs) and lipopolysaccharides has been suggested for health benefits. With increasing knowledge of the gut-brain-skin axis, further benefits for the skin have been suggested, although this warrants further investigation. Recent literature suggests that topical application of postbiotics may confer direct benefits for the skin. Of these, microbial lysates are widely characterized and are widely used in cosmetic products: *Bifidobacterium*/*Lactobacillus*/*Vitreoscilla filiformis* (*Vf*) [205,206,207,208,209,210].

#### 5.1.1. Probiotic Fractions

Oral probiotics have a positive effect on skin health, via the gut and the systemic route, by modulating the immune system [211].

For example, in addition to the ability to modulate both intestinal mucosal and systemic immune functions, the probiotic strain *Lactobacillus paracasei NCC2461* (*ST11*) conferred benefits to the skin to reinforce the skin barrier function and decrease skin sensitivity and dandruff conditions [210,212,213,214]. The probiotic *Lactobacillus johnsonii* La1 has been shown to protect the skin defenses by maintaining the number and function of Langerhans cells after UV exposure to the skin, as well as regulating skin inflammation [207,208,215]. In addition, probiotics have been shown to prevent and reduce the severity of atopic dermatitis, acne vulgaris, dry skin, prevent signs of photo-aging, and facilitate wound healing [211,216,217]. However, some published data raises safety concerns about the use of living probiotics in some specific contexts. A few weeks-gestation infants with abnormalities in their intestinal tract presented cases of bacteremia following *Lactobacillus GG* supplementation [218]. More recently, it has been demonstrated that Intensive Care Units patients that were administered with *Lactobacillus rhamnosus* as probiotics are at higher risk of developing bacteremia compared to those not receiving probiotics [219]. Thus, in the case of wounded or altered skin, special safety attention should be considered when cosmetics containing living probiotics are applied on the skin of people who might be critically ill.

Non-viable microorganisms extend the scope of probiotic concepts, for example, heat-killed and tyndallized probiotic lactic acid bacteria and *bifidobacteria* have key probiotic effects in gastrointestinal diseases [220]. Consumption of lactic acid bacteria extracts was found to have activity comparable to the live forms [221].

Health benefits on the skin can also be achieved by the topical application of inactivated probiotics [222,223]. The majority of topical bacterial extracts are ferments, ferment lysates, or ferment lysate filtrates generated after the cultivation and harvest of a probiotic microorganism.

Some bacterial extracts (*Bacillus coagulans*, *L. johnsonii*, *L. casei*, *L. plantarum*, and *L. acidophilus*) have antimicrobial properties that may support skin healing [224].

*Lactobacillus acidophilus* extract has been shown in vitro to scavenge reactive oxygen species following UVB-induced oxidative stress in keratinocytes [225].

*Lactobacillus plantarum* and *L. salivarius* lysates accelerated re-epithelisation by inducing keratinocyte migration [226].

Lysates of *L. rhamnosus* improved the skin barrier function in a reconstructed human epidermis and *S. thermophilus* extracts were able to increase ceramide production and improve skin hydration [227,228].

*Vitreoscilla filiformis* extract, via TLR2 activation, reinforced innate immunity and barrier function leading to a reduction of symptoms linked to atopic dermatitis and seborrheic dermatitis [205,206,229].

In reactive skin conditions, *B. longum* extract decreased skin sensitivity and improved resistance to physical aggression [209].

Topical inactivated probiotic fractions have also demonstrated efficacy in clinical trials in symptoms linked to acne, atopic dermatitis, and rosacea.

#### 5.1.2. Effector Molecules/Metabolites

The harmony of the microbiome ecosystem of the different human body sites is firmly associated with the molecules that they produce [230]. The human microbiome microorganisms produce metabolites and molecules acting on a diverse set of targets that can modulate many physiological functions in the host, among them the immune responses. Several acts as antibacterials, but many other products have unknown targets and effects on other commensals and the host. These molecules, playing as mediators of the microorganisms’ and microbes-host interactions, can have a non-local impact on tissues or organs where the microbiome is not established. This is the case for the gut microbiome-derived metabolites that could reach the bloodstream and modulate the homeostasis of the skin and its associated conditions. However, the skin differs from the gut in its physicochemical properties. It is a dry, acidic, lipid-rich, high-salt environment without exogenous nutrient sources, and therefore has low microbial biomass [9,231]. Microbes present on the skin—collectively referred to as the skin microbiota—are central to skin physiology and immunity. They produce a very rich array of metabolites/molecules.

Regardless of the origin of these postbiotic molecules, their beneficial effect on the skin is mediated by two major mechanisms:

#### 5.1.3. Modulation of the Skin Microbiome

Topical application of lactic acid-producing bacteria (LAB) lysates, in addition to their direct positive impact on the skin (anti-inflammatory, keratinocyte proliferation re-epithelization), has a protective impact against infection by pathogen bacteria *S. aureus* and *S. pyogenes*. Secretion of lactic acid was also proposed as the mediator of the beneficial effect on the skin (moisture retention of the skin) induced by the augmentation of *S. epidermidis* [232].

Another large family of postbiotic molecules, known for their protective property of the skin by restraining pathogenic bacterial infection, are bacteriocins [233].

Alpha-soluble modulins produced by *S. epidermidis* and free fatty acids (i.e., sapienic acid), which are the degradation products of lipids by bacteria, have antibacterial activity against *S. aureus* and various Gram(+) bacteria [234,235]. *S. epidermidis,* isolated from normal skin, unlike those from patients with AD, had antimicrobial activity against *S. aureus* via the secretion of antimicrobial peptides (AMPs). In vitro, the production of these peptides, namely lantibiotics such as epidermine and Pep-5, is dependent on the origin of the strain and inhibits *S. aureus* in a specific manner in synergy with LL-37 secreted by the keratinocytes [103]. *S. epidermidis* produces AMPs and PSMs which specifically inhibit the growth of *S. aureus* and group A streptococci [217]. *S. epidermidis* is also able to degrade *S. aureus* biofilms using the serine protease EspA in vitro [102]. The synergistic and specific action of human AMP LL37 and unknown AMPs produced by coagulase-negative *S. epidermidis* and *S. hominis* has specific antibacterial activity against the pathogen *S. aureus* involved in atopy [236]. In a Toll-like receptor (TLR)-3-dependent healing mechanism, *S. epidermidis* lipoteichoic acid and the lipopeptide LP78 reduced the inflammatory response to improve wound in a mouse model of skin injury. Additionally, some strains of *S. epidermidis* can diminish *S. aureus*–induced neutrophil recruitment and pro-inflammatory cytokine [18,237,238].

Transplantation of a mixture of *C. acnes* was successful after a one-week intervention period. Strains SLST types H1 + A1 + D1 did not show any adverse effect on the skin and the clinical relevance was not addressed in the study from [239]. *C. acnes* is capable of transforming glycerol into short-chain fatty acids, such as propionic acid and its derivatives which, on the contrary, inhibit the growth of USA300, a methicillin-resistant *S. aureus* [240,241]. Propionic acid finally helps to maintain pH value, which inhibits colonization by pathogenic microbes such as *S. aureus* [242]. *C. acnes* is known to produce specifically an enzyme called RoxP that possesses antioxidant activity and plays an important role in maintaining redox homeostasis on human skin [243].

Microbe-microbe interactions play a major role in the microbial ecosystem equilibrium and thus skin homeostasis. *S. epidermidis* limits the proliferation of C. acnes via the secretion of succinic acid and regulates the inflammation induced by C. acnes in the context of acne [244,245]. C. acnes, on the other hand, produce propionate, isobutyrate, and isovalerate which inhibit the formation of biofilms by *S. epidermidis* and increase its sensitivity to antibiotics [246]. In addition, C. acnes produces bacteriocins against S. epidermidis in vitro [247].

#### 5.1.4. Cross Talk with the Immune Response

The skin microbiota can induce and activate T lymphocytes, both in the basal state and during infection. This interaction with the immune system helps to control skin homeostasis and protect the immune system against pathogens such as *Leishmania major* and *Candida albicans* [248]. It is well known that cellular mediators produced by *S. epidermidis* modulate the production of various cytokine IL17+CD8+T Cells [249]. LTA lipoteichoic acid is a major component of the cell wall of gram positive bacteria such as *S. epidermidis* and also a ligand for TLR2. Although mechanism-dependent on the latter, it has been identified to have an anti-inflammatory effect on keratinocytes and to stimulate the production of keratinocyte stem cell factor (SCF). It is also critical for mast cell differentiation [18,250]. TLR2 and the production of pro-inflammatory cytokines induce the recruitment and maturation of T lymphocytes in CD4 + and CD8 which protect against skin infections and favor healing. Finally, the activation of TLR2 (especially via lipoteichoic acid) suppresses the inflammation mediated by TLR3 [15,251,252].

SCFAs from *C. acnes* modulate cytokine expression and may influence both the local pilosebaceous unit as well as the surrounding skin. These SCFAs inhibit the activity of keratinocyte histone deacetylase, an enzyme involved in epigenetic control, resulting in enhanced sensitivity to TLR activation and cytokine expression by keratinocytes and sebocytes [253]. Certain clinical strains of *C. acnes* induce the expression of hBD2 by NHEK keratinocytes via the activation of the TLR2 and TLR4 receptors and the culture supernatant of *C. acnes* (ATCC6919) induced expression of hBD2 and LL-37 mRNAs by keratinocytes (HaCat) [254,255]. Similar results have been published for different species of *Malassezia* [256,257].

Another bacterial metabolite family of interest in the cross-talk between bacteria and the host is indolic metabolites. The tryptophan-derived Indole 3 aldehyde was shown to reduce inflammation by binding to the Aryl hydrocarbon receptor AhR [258].

In many cases, metabolite-host interaction is modulated by the immune system. This is the case via the MAIT cell system, which is known to recognize riboflavin (B2) intermediates which are metabolites produced specifically by the major microorganism genera components of the skin [259]. Therefore, MAIT cell-specific immunity through recognition of riboflavin biosynthesis intermediates is likely to play an important role in skin barrier health.

In addition, some skin bacterial metabolites may trigger specific actions on pathways that do not trigger inflammation. This is the case of the nucleobase analog 6-N-hydroxyaminopurine (6-HAP) which is produced by *S. epidermidis* and is known to have selective antiproliferative action against tumor cell lines in vitro [22].

Microbial effector metabolites also play a role in wound healing. Traditionally, treatment of wounds, ulcers, and burns has been paralleled with antibiotic treatment. However, in recent years, a paradigm shift has taken place following studies demonstrating better wound healing in the presence of bacteria [260]. Pathogenic bacteria are thought to delay healing [261,262] while interactions between the commensal microbiota and the skin can aid wound healing by regulating the immune response and promoting repair of the skin barrier [15,263]. For example, the interaction of lipoteichoic acid (LTA) from *S. epidermidis* with TLR2 in keratinocytes inhibits inflammatory responses and therefore limits tissue damage and promotes wound healing [18,264].

## 6. Future Implications/Outlook

Postbiotics, including probiotic fractions and effector molecules, are solutions already used in the dermo-cosmetic field; however, the ambition for the cosmetic industry is to add live probiotics to cosmetic formulas with the expectation of potentially higher performance that would probably be driven by a dialogue between added living-microbes and host cells.

However, the use of probiotics as cosmetic ingredients raises many questions. From a formulation standpoint, the first challenge is to maintain these microorganisms alive in a cosmetic formulation. Most cosmetics are water-based and pH neutral or slightly acidic, which can be considered as favorable conditions, but they also contain some ingredients that could affect probiotics ‘survival’: surfactants, chelating agents, glycols, preservatives, fragrance. Moreover, the preservation of cosmetic products from microbial contamination and proliferation is a safety and regulatory requirement. Therefore, the challenge is to maintain probiotics alive in cosmetic products while preventing the growth of microorganisms that could adversely affect the health of the consumers. This can be achieved by different means such as the encapsulation of probiotics, or the use of suitable packaging where the living bacteria are kept separately and mixed with the formulation at the time of use.

From a regulatory standpoint, the ICCR report indicates that “There were no unique regulations governing cosmetic products or ingredients intended to work specifically with the skin’s (or mucosal) commensal microbiome. Rather, such products are subject to the applicable rules and regulations governing cosmetics in each respective jurisdiction, including those covering both safety and product representation (i.e., claims). Several jurisdictions pointed out that while no distinct regulations exist specific to these products there are general quality standard requirements such as microbiological limits which apply to all cosmetic products, including those containing live or viable microorganisms” [204].

The microbiological limits for cosmetic products are given in an International Standardization Organization (ISO) standard [265]. This document states that although cosmetics are not required to be sterile, microorganisms present in a product should not cause an adverse effect on consumer safety or product quality. Therefore, the manufacturer must respect the Good Manufacturing Practices and take the necessary precautions to limit the introduction of microorganisms from raw materials, processing, and packaging.

In this standard, microorganisms are considered as contaminants that are unintentionally introduced in the cosmetic product and microbiological limits are established to ensure product quality and consumer safety. Therefore, those limits should not apply to probiotics which are well-characterized microorganisms intentionally introduced in cosmetic products to achieve a cosmetic benefit. However, discussions are still ongoing, and some clarification is needed to allow the use of living bacteria as cosmetic ingredients.

The use of live probiotics is one of the major future applications in the dermo-cosmetic industry but not the only one. Each human has his or her own ‘microbial fingerprint’ that is specific to his or her skin and this specific microbiome may influence its homeostasis. The microbiome is the path to an individualized skincare routine.

In this perspective, personalized microbiome-derived cosmetic solutions that would intervene specifically are the future paradigm for safe, effective, and successful skin/scalp care products.

## 7. Conclusions

**Figure 2:**
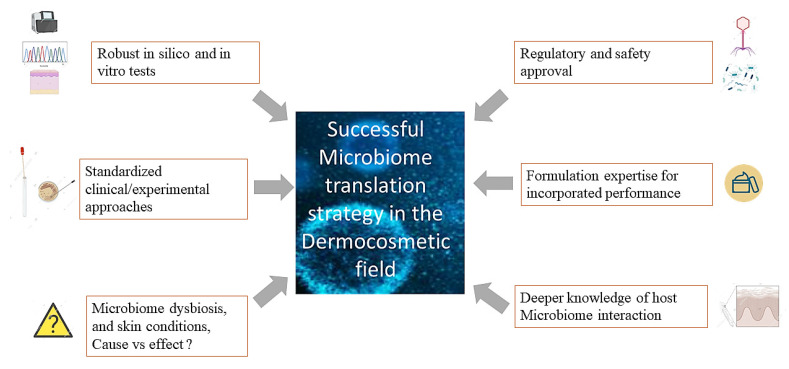
Resuming the best strategy for a successful translation of Microbiome-based concepts into cosmetic products of the future. Combined approaches of multi-omics technologies, powerful data mining tools, and representative 3D vitro skin models associated with standardized and unbiased experimental approaches dedicated to skin Microbiome analysis are key. Harnessing recent scientific breakthroughs and deciphering the famous causality question allied to better characterization of the interaction between the microbiome, the immune system and skin cells in various skin conditions would accelerate the translation. Finally, consideration of regulatory and safety aspects related to these new/targeted Microbiome-derived technologies (postbiotics, phages, probiotics…) and how to leverage their performance in different formulation types is essential.

Research is at the dawn of a «new generation» cosmetic that will use the skin’s microbiome to provide lasting products with new efficient performance. To bring to light this rising cosmetics category that harnesses the potential of the cutaneous microbiome, it is essential to dissect the dynamic interactions existing between microorganisms and the interplay host/Microbiome. Researchers would also need to understand the regulatory/safety framework to translate these innovations (Figure 2). However, only rigorous and unbiased experimental approaches considering the specificity of the skin-microbiome environment can be applied. This discovery will be made possible by coupling multi-omics technologies, statistical data mining, and representative 3D skin models. These approaches may provide the opportunity to establish microbiome/skin condition causality and, subsequently, cosmetic solutions. The consideration of subtle regulatory environments and country-specificities will also be of high concern.

## Figures and Tables

**Table 1:** Major taxonomic modifications of the skin/scalp microbiome in skin diseases.

Skin Condition	Microbiome Shift	Reference
Atopic Dermatitis (AD)	Decreased microbial diversity Increase in *S. aureus* associated with disease severity and increase in *S. epidermidis* during flares Decrease in *C. acnes, lactobacilli*, and *Burkholderia* spp. Increase in *M. sympodialis*, (and secondly *M. globosa*, *M. dermatis*, *M. restricta*)	[75,76,77,78,79,80,81]
Psoriasis	Increase in *C. simulans, C. kroppenstedtii, Finegoldia* spp., and *Neisseriaceae* spp. Decrease in *C. acnes, lactobacilli, and Burkholderia* spp. No difference in the amount of *Malassezia* between lesion area of psoriasis and healthy skin (measured by PCR) but there is more diversity of *Malassezia* species in patients with psoriasis compared to healthy individuals Increase in *Brevibacterium, Kocuria palustris*, *Gordonia,* and increase in *M. restricta* (back) and *M. sympodialis* (elbow) increased in psoriatic lesions.	[76,79,82,83]
Acne	Increase in the proportion of *C. acnes* strains presenting with virulence factors Increase in *S. epidermidis* (secondary to *C. acnes*) *Malassezia* spp. could be involved in the development of acne	[46,84,85,86,87,88]
Rosacea	Increased in Demodex mites on the skin.	[89,90]
Vitiligo	Decrease in bacterial diversity	[74]
Seborrheic dermatitis (SD) and Dandruff	Increase in *S. epidermidis* and decrease in *C. acnes*. Increase in the population of *Malassezia restricta* and *Malassezia globosa* on the scalp. *Malassezia* spp. metabolize and oxidize sebum-derived lipids into inflammatory compounds and produce indole derivatives (malassezin, indolocarbazole) which may impact skin inflammation through aryl hydrocarbon receptors.	[88,91,92,93,94]

**Table 2:** Resuming the cosmetic description of prebiotic and postbiotic ingredients.

Postbiotic (Including Probiotic Fraction or Extract)	Prebiotic
Non-viable ingredients comprised of inactivated microorganisms and/or soluble factors (products or metabolic by-products) released by live or inactivated microorganisms, added to a cosmetic product to achieve a cosmetic benefit at the application site, either directly or via an effect on the existing microbiota. Categories: 1/Ferments, lysates, extracts, filtrates, 2/Non-viable microorganisms (inactivated/heat-killed), 3/Metabolic products/by-products (isolated)	Non-viable ingredients are added to a cosmetic product to be actively used as nutrients by the microbiota of the application site to achieve a cosmetic benefit. Examples: ingredients such as fibers, sugars, minerals, but also complex biological mixtures/extracts, etc.

## Data Availability

No new data were created or analyzed in this study. Data sharing is not applicable to this article.
